# Obesity and the receipt of influenza and pneumococcal vaccination: a systematic review and meta-analysis

**DOI:** 10.1186/s40608-016-0105-5

**Published:** 2016-05-03

**Authors:** John A. Harris, Michelle H. Moniz, Brad Iott, Robyn Power, Jennifer J. Griggs

**Affiliations:** Department of Obstetrics and Gynecology, University of Michigan, 2800 Plymouth Road, Building #10 Room G016, Ann Arbor, MI 48109-2800 USA; Division of General Internal Medicine, Department of Internal Medicine, University of Michigan, 2800 Plymouth Road, Building #10 Room G016, Ann Arbor, MI 48109-2800 USA; Division of Hematology and Oncology, Department of Internal Medicine, University of Michigan, 2800 Plymouth Rd, Building 16 Room 400S, Ann Arbor, MI 48109-2800 USA

**Keywords:** Obesity, Vaccination, Influenza, Pneumococcus, Systematic Review, Meta-analysis

## Abstract

**Background:**

Obesity is a risk factor for inadequate receipt of recommended preventive care services. The objective of this study was to assess the relationship between increasing body mass index and receipt of influenza and pneumococcal vaccinations. A systematic review of the PubMed, Embase, and Web of Science databases was conducted from January 1966 to May 2015 for cohort and cross-sectional studies that assessed the relationship between body mass index and the receipt of vaccinations for influenza and pneumococcus. Separate meta-analyses by obesity classification were performed using a random effects model.

**Results:**

Six cross-sectional and three cohort studies were included. Average vaccine uptake was 50.4 % for influenza vaccination and 34.6 % for pneumococcal vaccination. Compared to normal weight patients, combined odds ratio (95 % confidence interval) for influenza vaccination was 1.11 (95 % CI 0.97–1.25) for obese (≥30 kg/m^2^) patients. When the outcome was reported by obesity class, combined odds ratios of influenza vaccination were 1.13 (95 % CI 1.02–1.24) for Class I (30–34.9 kg/m^2^) obesity, 1.21 (95 % CI 1.05–1.37) for Class II obesity (35–39.9 kg/m^2^), and 1.19 (95 % CI 0.95–1.42) for Class III obesity (≥40 kg/m^2^) patients. Compared to normal weight patients, combined odds ratio of pneumococcal vaccination were 1.20 (95 % CI 1.13–1.27) for obese patients. When the outcome was reported by obesity class, combined odds ratios were 1.08 (95 % CI 1.04–1.13) for Class I obesity patients, 1.13 (95 % CI 1.10–1.16) for Class II obesity patients, and 1.26 (95 % CI 1.15–1.38) for Class III obesity patients for pneumococcal vaccination.

**Conclusions:**

Combined findings from the current literature suggest that adults with obesity are more likely than non-obese peers to receive vaccination for influenza and pneumococcus. However, suboptimal vaccination coverage was observed across all body sizes, so future interventions should focus on improving vaccination rates for all adults.

**Electronic supplementary material:**

The online version of this article (doi:10.1186/s40608-016-0105-5) contains supplementary material, which is available to authorized users.

## Background

The World Health Organization (WHO) Strategic Advisory Group of Experts (SAGE) on Immunization and the US Centers for Disease and Control and Prevention (CDC) recommends vaccination of eligible adults for influenza virus and streptococcus pneumoniae bacteria. Influenza vaccination is recommended yearly for adults with few contraindications [1–3]. For instance, pneumonia vaccination is recommended by the CDC for all persons over 65 years old, as well as for younger adults with select medical risk factors (e.g. diabetes mellitus, chronic heart disease, chronic lung disease, chronic liver disease, chronic renal disease, cancer, HIV infection, etc) [4]. Influenza vaccination decreases the rate of diagnosed influenza and the severity of influenza morbidity and mortality, particularly in persons with comorbid diseases [5–7]. Similarly, vaccination against streptococcus pneumoniae bacteria effectively prevents laboratory-confirmed pneumonia infections and invasive pneumococcal disease [8–10].

Despite the many benefits of vaccination and their strong endorsement from major national professional organizations, vaccine uptake is lower than desired. For example, in the US, the median vaccination rate is 60.7 % for influenza and 70.0 % for pneumococcal vaccination among adults in 2011, compared to US Healthy People 2020 goals for influenza (70 %) and pneumococcal (90 %) vaccination of eligible adults [8, 9]. Many factors are known to affect the receipt of vaccination, including age, race, personal beliefs, and comorbid conditions [10–12]. Persons with chronic illnesses suffer disproportionate morbidity and mortality from vaccine-preventable illness; therefore, there is particular interest in ensuring vaccine coverage of these especially vulnerable adults.

As with many other chronic diseases, obesity is associated with increased mortality from both influenza and pneumococcal infections [13–15]. Obesity – which affects approximately one third of US adults and 600 million people worldwide – may impact a person’s receipt of preventive health services, but the direction of this association is unclear [16, 17]. In the case of cancer screening, increasing levels of obesity in women are associated with lower rates of breast and cervical cancer screening [18, 19]. However, studies in Medicare and Veteran’s Administration populations have documented higher rates of vaccination in obese patients [20] The purpose of this systematic review and meta-analysis is to determine whether increasing body mass index is associated with less likelihood of adults receiving indicated influenza and pneumococcal vaccinations in cohort and cross-sectional studies.

## Methods

### Search strategy and selection criteria

We adhered to the Preferred Reporting Items for Systematic Reviews and Meta-Analyses (PRISMA) and Guidelines for Meta-Analyses and Systematic Reviews of Observational Studies (Additional file 1) recommendations in conducting this systematic review. The study was deemed exempt from review by the University of Michigan Institutional Research Board – Medical. PubMed (January 1966 to May 2015), EMBASE (January 1980 to May 2015), and Web of Science (January 1900 to May 2015) databases were searched using the preassigned search terms. We reviewed the cited references of the included studies for additional studies. Reviewers included studies that compared vaccination rates for influenza and pneumococcus by patient obesity where the predictor of interest was patient body mass index, and the outcome of interest was influenza or pneumococcal vaccination.

We developed a detailed search protocol with the help of a medical librarian. Full details of the search strategies are noted in the Appendix 1. In brief, our search included controlled vocabulary and keywords similar to “obesity” and “streptococcus pneumococcal or influenza virus vaccination,” and the outcome “receipt of vaccination”. We included prospective or retrospective cross-sectional or longitudinal cohort studies. Only articles published in English were included. The search and review was last updated in May 2015. The study protocol was not eligible for registration with PROSPERO International prospective register of systematic reviews due to the outcome being process-related rather than intervention-related [21].

### Study selection

All article titles and abstracts were reviewed for inclusion by two independent reviewers (BI, RP, or JH) using a template defining inclusion and exclusion criteria. The inclusion criteria for the first screening were: a population including comparison of non-obese and obese adults, the receipt of vaccination for influenza or pneumococcus as a recorded outcome and identification as a cohort or cross-sectional study. The inclusion criteria for the full-text, second screening used the same inclusion criteria as the first. Discrepancies in any of the reviews were adjudicated by consensus.

### Data extraction and quality assessment

The same two independent reviewers examined the full text of the study, assessing for the protocol inclusion and exclusion criteria. Human papilloma virus was included in the search terms, but no studies met inclusion criteria; we are therefore unable to include this vaccine in the analysis. The study team reviewed all studies meeting criteria for relevant information and results were extracted using a data collection template. Abstracted data included detailed information about the study objectives, design, participants, covariates, outcomes, statistical methods, and a quality assessment.

We assessed methodological quality using the US National Institute of Health National Heart, Lung, and Blood Institute Quality Assessment Tool for Observational Cohort and Cross-Sectional Studies [22]. This tool measures 14 different criteria which are then used to give each study an overall quality rating of good, fair, or poor.

### Data synthesis and statistical analysis

We created tables to summarize the study populations, inclusion and exclusion criteria, vaccination type, and outcomes. The principal summary measure was odds ratios. We performed a meta-analysis in studies that reported body mass index in the categories defined by the World Health Organization and the National Institutes of Health (normal: body mass index (BMI) 18.5–24.9 kg/m^2^, obesity BMI ≥ 30 kg/m^2^, class I obesity BMI 30–34.9 kg/m^2^, Class II obesity BMI 35–39.9 kg/m^2^, Class III obesity BMI >40 kg/m^2^) or between normal weight and obesity ≥30 kg/m^2^ if that was the only comparison reported.

We used random-effects (DerSimonian and Laird) models to calculate odds ratios and 95 % confidence intervals for pneumococcal and influenza vaccination [23]. For studies reporting adjusted relative risk or predicted prevalence, we calculated odds ratios with normal body mass index as the reference category. We calculated an I^2^ statistic to test for heterogeneity, and the Begg’s test and visual inspection of the funnel plot to assess for the possibility of publication. We used STATA 13.0 (StataCorp, College Station, TX) for all analyses. The study was deemed exempt by The University of Michigan Medical Institutional Review Board (HUM00097519).

## Results

**Fig. 1:**
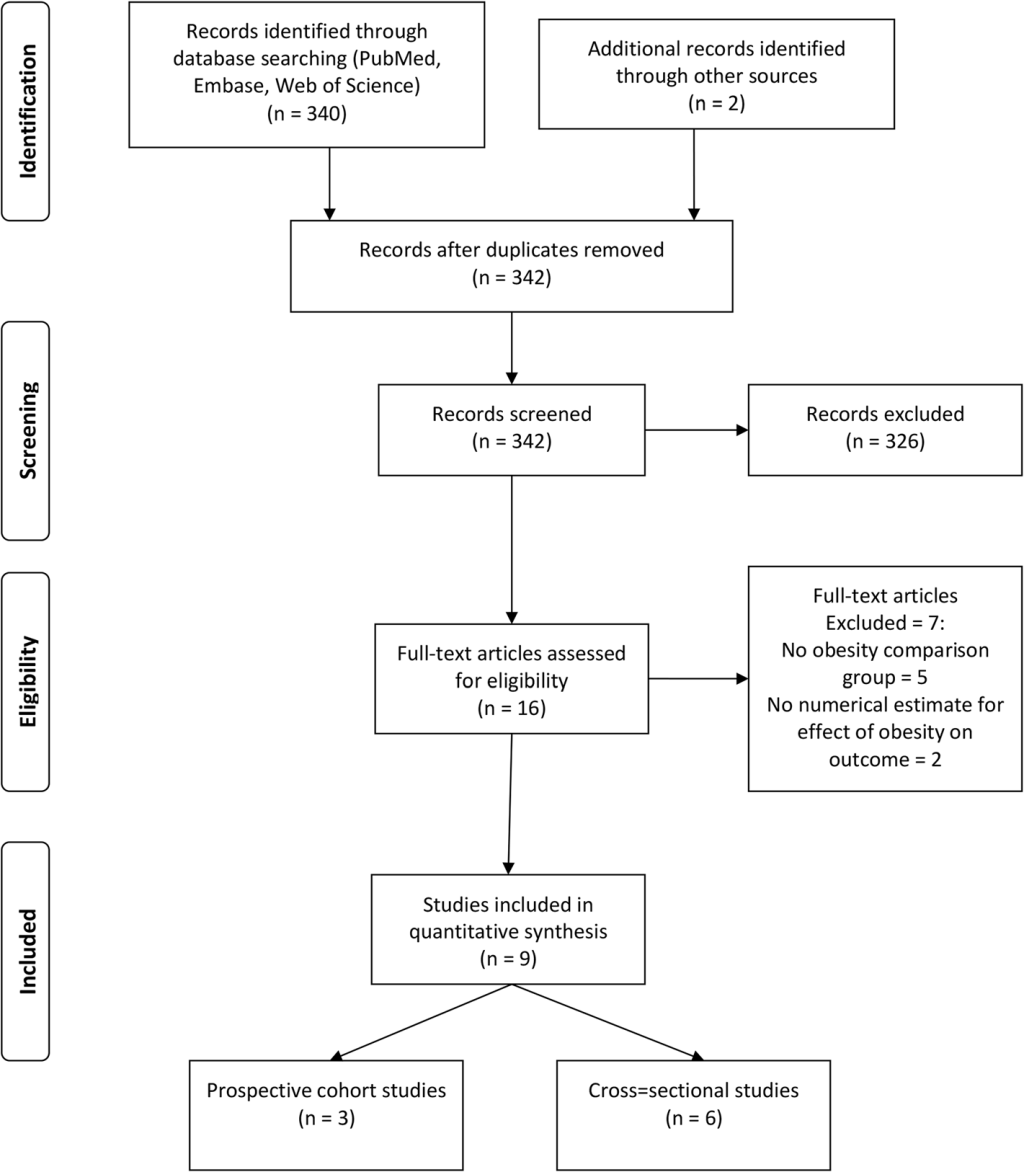
Study flow diagram

Of 342 titles identified in the full search of three databases and hand searching, nine articles met inclusion criteria (Fig. 1).

### Obesity classifications

Among included studies, obesity was most frequently measured by BMI, which was most often defined as underweight (BMI < 18.5 kg/m^2^), healthy weight (BMI 18.5–24.9 kg/m^2^), overweight (BMI 25–29.9 kg/m^2^), obese (BMI ≥30 kg/m^2^) [20, 24–27] or defined by World Health Organization cutoff points as underweight (BMI < 18.5 mg/m^2^), healthy weight (BMI 18.5–24.9 kg/m^2^), overweight (BMI 25–29.9 kg/m^2^), obesity Class I (BMI 30–34.9), obesity Class II (BMI 35–39.9 kg/m^2^), obesity Class III (BMI ≥40 kg/m^2^) [28–32]. A single study defined obesity as a BMI ≥30 kg/m^2^ or waist circumference >102 cm in men and >88 cm in women [26]. The reference population for comparison was generally the normal weight category; in one case overweight and normal weight and underweight were grouped together and used as the reference category [33].

### Outcome definitions

Influenza vaccination was defined most often as receiving vaccination within the past 12 months or past year. Single studies defined influenza vaccination as receipt within the past 2 years or 4 out of the past 5 years (and at least 8 months apart) [31, 32]. Pneumococcal vaccination was defined most often as receiving vaccination at least once in the participant’s life. A single study defined pneumococcal vaccination as receipt within the past 5 years [25].

### Study characteristics

Study populations involved one of two groups: 1) adults aged 18 or older (commonly for influenza vaccination studies) and 2 adults aged 65 years or older (commonly for pneumococcal vaccination studies). All studies examined both men and women in population-based (between 3000 and 1 million participants) national or international settings. Six studies were cross-sectional, three were cohort studies, and all studies included data for was from population-based surveys, including the Behavioral Risk Factor Surveillance System, Medical Expenditure Panel Survey, Health and Retirement Study, Medicare Current Beneficiary Survey, and the Survey of Health, Aging, and Retirement in Europe. BMI and vaccination information was generally self-reported except for studies using Veteran’s Health Administration (VA) populations, where these data represent measure biometric indices and vaccination variables abstracted from the electronic medical record [20, 32].

### Quality assessment

Using the United States National Institute of Health (NIH) National Heart, Lung and Blood Institute (NHLBI) Quality Assessment Tool for Observational Cohort and Cross-Sectional Studies, we rated 4 studies at a “good” quality rating and 5 studies at a “fair” quality rating. The studies all used well-designed surveys except studies using VA or Medicare administrative datasets. The number and type of covariates differed widely among the studies; use of a more complete list of model covariates was a key differentiating characteristic of studies rated “good”.

### Confounder covariates

Most studies included a wide variety of covariates in their regression models. Common covariates included age, sex, race, ethnicity, education level, tobacco and drug use. Measures of prior healthcare utilization were included in five studies [20, 24, 27, 30, 31]. Utilization measures varied from time since last routine check-up to ever having received a mammogram to number of ambulatory visits in the past year.

### Study outcomes

**Fig. 2:**
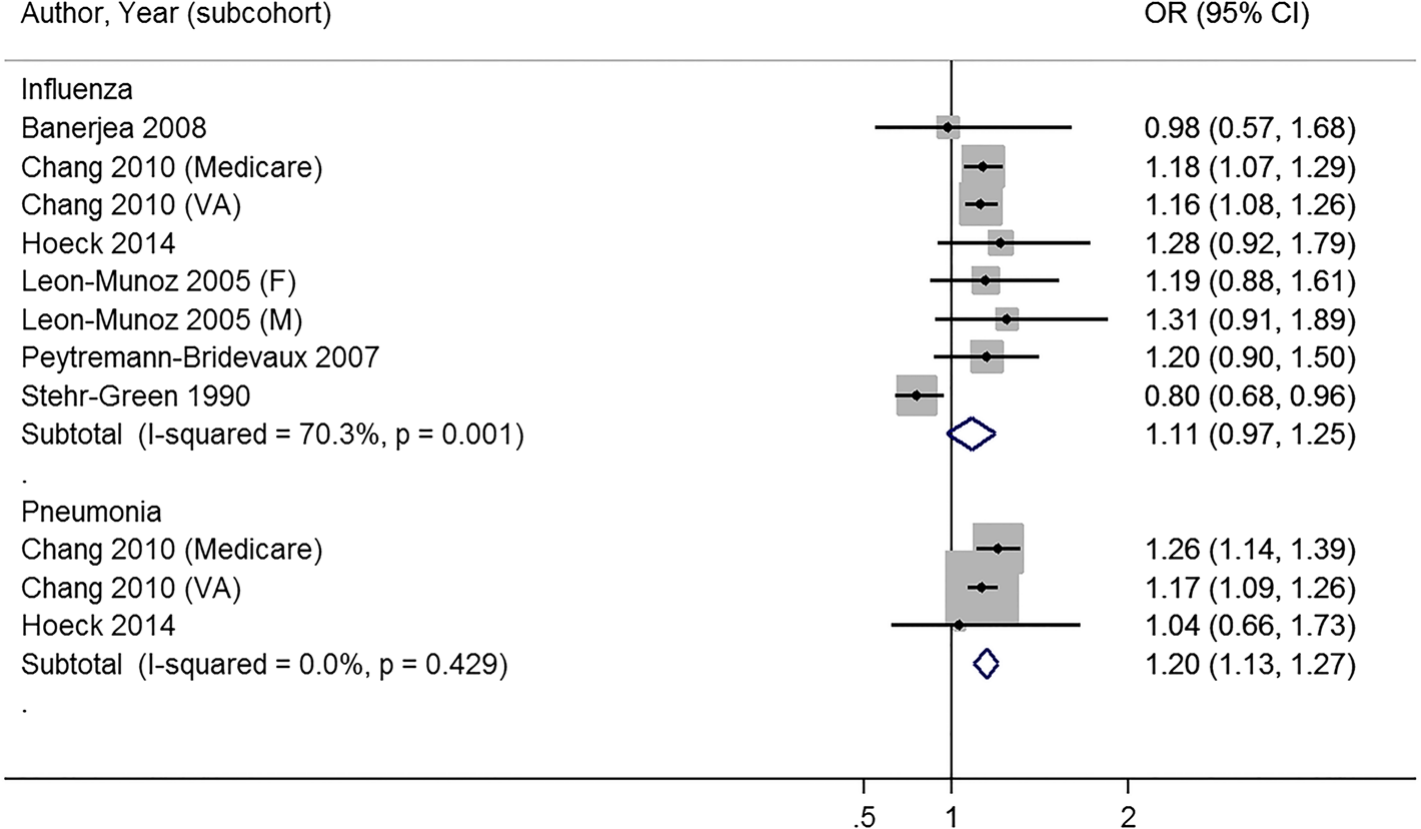
Meta-analysis of studies on receipt of influenza and pneumococcal vaccination with body mass index categorized by normal weight and obese (≥30 kg/m^2^)

**Fig. 3:**
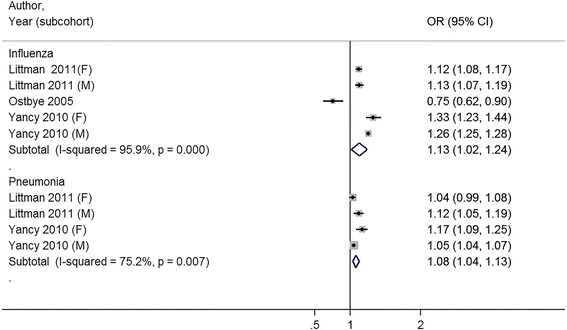
Meta-analysis of studies on receipt of influenza and pneumococcal vaccination with body mass index categorized by Normal Weight and Class I Obesity (30–34.9 kg/m^2^)

**Fig. 4:**
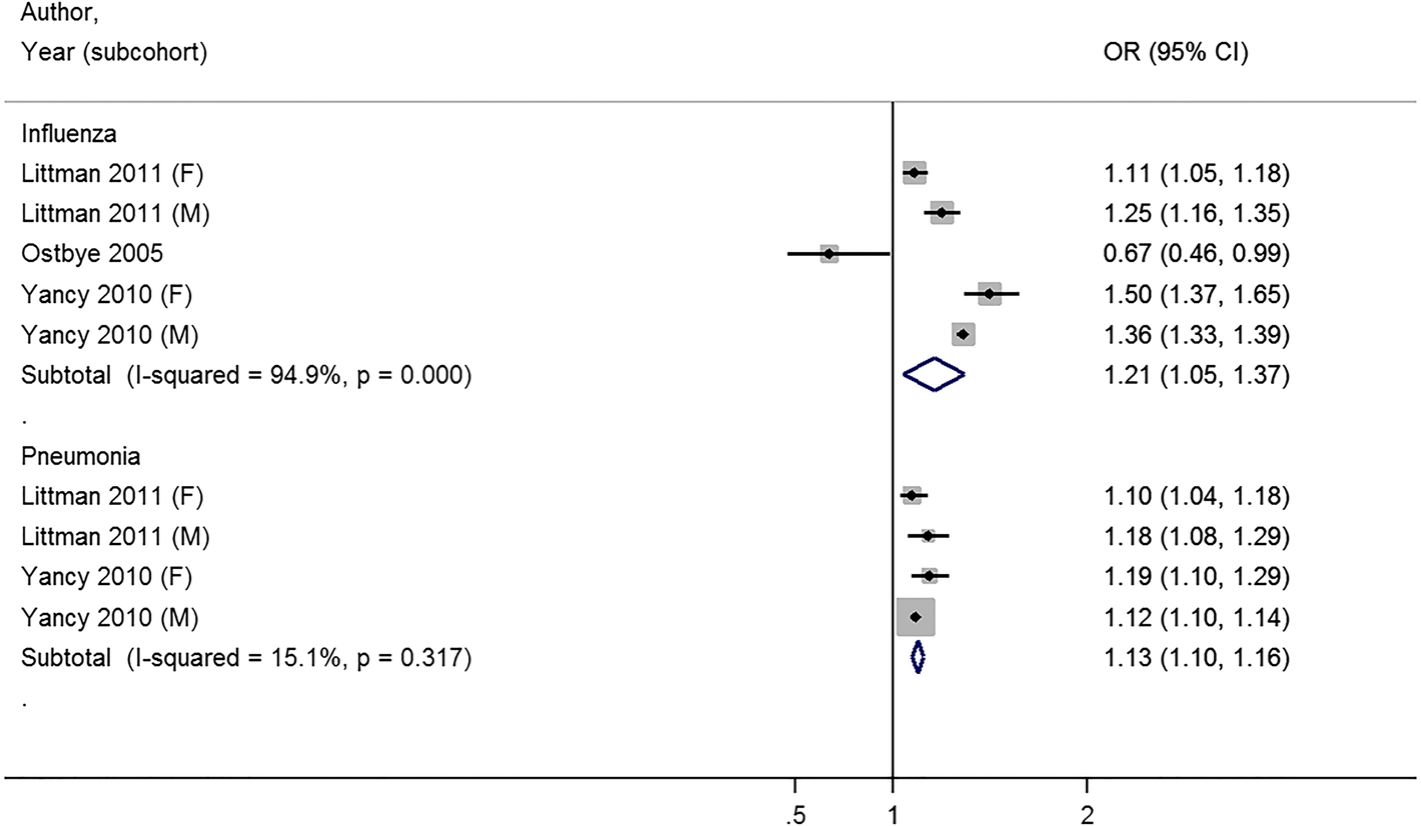
Meta-analysis of studies on receipt of influenza and pneumococcal vaccination with body mass index categorized by Normal Weight and Class II Obesity (35–39.9 kg/m^2^)

**Fig. 5:**
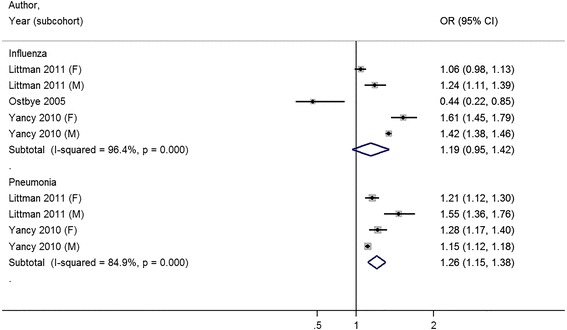
Meta-analysis of studies on receipt of influenza and pneumococcal vaccination with body mass index categorized by Normal Weight and Class III Obesity (≥40 kg/m^2^)

The full study characteristics are presented in Table 1. The general vaccination rate for influenza ranged from 21.1 % [32] to 72.2 % [31]; the average vaccine uptake was 50.4 %. The general vaccination rate for pneumococcus ranged from 13.4 % [25] to 49.5 % [20]; the average vaccine uptake was 34.6 %. Of nine studies, three studies reported a statistically significant association between increasing BMI and receipt of vaccination [20, 30, 32], four studies were unable to reject the null hypothesis that there was no association between increasing BMI and receipt of vaccination [24–27], and two studies reported a statistically significant inverse association between increasing BMI and receipt of vaccination [31, 33]. Compared to normal weight patients, the combined odds ratio for influenza vaccination was 1.11 (95 % CI 0.97–1.25) for obese (≥30 kg/m2) patients (Fig. 2) . When the outcome was reported by obesity class, combined odds ratios were, 1.13 (95 % CI 1.02–1.24) for class I obesity patients, 1.21 (95 % CI 1.05–1.37) for class II obesity patients, and 1.19 (95 % CI 0.95–1.42) for class III obesity patients for influenza vaccination (Figs 3, 4 and 5).

**Table 1:** Results for studies included in qualitative and quantitative analysis

Author (year)	Year	Outcome Measure	Study Size	Risk Adjustment Covariates	Overall Rate of Vaccination	NIH NHLIBI Quality Rating
Banerjea et al. [24]	2008	Influenza vaccination in last year	4299	Age, race/ethnicity, marital status, education, employment, income, access to health care, contact with healthcare system, medical conditions, health status, comorbid chronic conditions, mental illness	55.6 %	Good
Chang et al. [20]	2010	Influenza vaccination in last year; Pneumo-coccal vaccination ever	Influenza: 33071 (Medicare), 28337 (VA), Pneumo-coccal: 32266 (Medicare), 28337 (VA)	Age, sex, race/ethnicity, marital status, education, income, self-rated health, quartile of clinical complexity, quartile of visit frequency, year of receipt, age squared term, interaction between weight and sex, weight and race	Influenza: 65.7 %; Pneumo-coccal: 49.5 %	Good
Hoeck et al. [25]	2014	Influenza vaccination in past year; Pneumo-coccal vaccination within last 5 years	4544	Age, gender, highest level of education, living situation, region, self-assessed health, longstanding health issues, household income, risk factors, health status	Influenza: 63.1 %; Pneumo-coccal: 13.4 %	Fair
Leon-Munoz et al. [26]	2005	Influenza vaccination in last 1 year	2919	Age, educational level, size of place of residence, tobacco, alcohol	62.8 %	Fair
Littman et al. [30]	2011	Influenza vaccination in past year; Pneumo-coccal vaccination ever	Influenza: 537138, Pneumo-coccal: 411039	Age, education, race/ethnicity, self-reported personal doctor, healthcare coverage, time since last routine checkup	Influenza: 51.1 %; Pneumo-coccal: 41.0 %	Fair
Ostbye et al. [31]	2005	Influenza vaccination in the last 2 years	10588	Age, gender, race, education, birth country, marital status, household income, insurance, smoking, exercise, self-reported health, diabetes, heart disease, cognitive impairment, hospitalization in last year, number of outpatient visits, survey wave	72.2 %	Good
Peytremann-Bridevaux et al. [27]	2007	Influenza vaccination in last year	13859	Age, gender, marital status, years of education, purchasing power parity-household income adjusted for size of household, smoking status, excessive alcohol consumption, country of residence, diagnosis of hypertension, heart disease, diabetes, cholesterol, arthritis, reported number of ambulatory visits	50.9 %	Good
Stehr-Green et al. [33]	1990	Influenza vaccination in last year	9799	Gender, race, education, income, employment outside home, seat belt use, smoking, smokeless tobacco, alcohol use, drinking and driving, sedentary lifestyle, medical exam in last year, hypertension, ever had mammogram	32 %	Fair
Yancy et al. [32]	2010	Influenza vaccination 4 out of 5 most recent years, at least 8 mo apart for 65+ or high risk; Pneumo-coccal vaccination once for 65 years old or older or high-risk under 65 year olds	1058599	Age, gender, race/ethnicity, marital status, outside insurance, healthcare eligibility, geographic region, primary care provider provider, health status (algorithm for Charlson comorbidity score)	Influenza: 21.1 %; Pneumo-coccal: 32.8 %	Fair

*BMI* Body Mass Index, *VA* Veterans Administration

Among the three studies with NIH NHLBI Quality Assessment Tool “good” ratings, where model covariates included measures of outpatient utilization, one study each that found a positive or negative association, and two studies found a statistically insignificant association between receipt of vaccination and increasing body mass index [20, 24, 27, 31].

Among studies that measured pneumococcal vaccination, three of four studies noted a positive association between receipt of vaccination and increasing body mass index [20, 30, 32]. Compared to normal weight patients, the combined odds ratio for pneumococcal vaccination was 1.20 (95 % CI 1.13–1.27) for obese patients. When the outcome was reported by obesity class, combined odds ratios were 1.08 (95 % CI 1.04–1.13) for class I obesity patients, 1.13 (95 % CI 1.10–1.16) for class II obesity patients, and 1.26 (95 % CI 1.15–1.38) for class III obesity patients for pneumococcal vaccination (Figs. 2, 3 and 4).

Visual inspection of the funnel plots did not suggest publication bias, but in many of the comparisons there were few studies to compare. Begg’s test for publication bias was insignificant except for pneumococcal vaccination and Class I and III obesity (*P* = 0.04 and *P* = 0.04) (Appendix 2).

## Discussion

This systematic review and meta-analysis identified nine studies evaluating the association between patient body mass index and receipt of influenza and pneumococcal vaccination. It suggests that patients with obesity overall are more likely than patients without obesity to receive vaccination for both influenza and pneumococcus.

A variety of patient-level and systems-level factors may explain this finding. Patient level factors such as perceived susceptibility to disease and perceived vaccine effectiveness are predictors of vaccine acceptance [34, 35]. Adults with obesity may be more likely to perceive higher risk from vaccine-preventable diseases and more accepting of the benefits and safety of recommended vaccinations. As patients with obesity are frequently counseled about the risks of obesity-related disease, these patients may perceive a higher personal risk of future disease than non-obese patients.

Even as this study finds that increasing body mass index was associated with increasing rates of vaccination for influenza and pneumococcus, the overall rate of vaccination observed is still very low. Only half of the included studies reported vaccine uptake greater than 50 %. Large percentages of all adults, both obese and non-obese are still under-vaccinated, so there is still much progress to be made in people of all body sizes to reach these reasonable vaccination uptake goals [9].

The study populations in this systematic review vary significantly by age, sex, and race. Some studies include only older adults, due to the age-related recommendations for pneumococcal vaccination, while others include the full general adult populations. The effect of comorbid illness like obesity may interact with age to affect the receipt of preventive services like vaccinations. Analogous studies in cancer screening have noted decreased rates of screening as age increases, but not as comorbidities increase [36–38]. Unlike non-obese populations, as obese patient’s age, they may be more likely to be identified for preventive health services. For instance, obesity and diabetes are closely associated, and diabetes is an indication for vaccination for both influenza and pneumococcus. This may explain why older obese adults included in this analysis were more likely than non-obese peers to receive pneumococcal vaccination. In addition, sex, race, and ethnicity are all associated with variations in the utilization of preventive care services like colorectal cancer screening [39, 40]. Age, sex, and race differences within the included studies may explain some of the variation in effect size an effect directionality noted in the meta-analysis.

Systematic reviews and meta-analyses of breast, cervical and colon cancer screening document variation in the relationship between obesity and cancer screening, but overall suggest a small but persistent inverse relationship between increasing BMI and decreasing cancer screening uptake [18, 19, 41, 42]. The current study’s findings may be different due to the complex and multiple factors that influence uptake of preventive services in persons with obesity.

The current study’s converse finding that obesity is associated with increased vaccination may reflect the complex factors that affect the receipt of preventive care services in patients with obesity. Vaccination is a very different type of preventive care service than cancer screening. Vaccination is a relatively simple procedure that may be performed during almost any outpatient or inpatient interaction. Patients stay fully dressed, and the procedure may be performed by nursing personnel with minimal physician oversight. Cancer screening for breast and cervical cancer are presently much more invasive and complex procedures. Patients with obesity have reported multiple barriers to cancer screening including provider stigma, personal discomfort with the procedures, and perceived disrespectful communications with healthcare providers and staff [41, 43, 44]. Finally, cancer screening is more subject to issues of overuse which may complicate the interpretation of studies on the receipt of cancer screening; overuse of vaccination services is rarely a concern, which simplifies interpretation of studies on the receipt of vaccination services.

This study, together with previous findings in cancer screening, suggests that patient body mass index should be examined as a part of any evaluation of the receipt of primary preventive services. Whether obesity is a risk factor for underuse, as in cancer screening, or for overuse, as in influenza and pneumococcal vaccination, both trends are a potential problem for health care delivery systems. These preventive care inefficiencies experienced by patients with obesity may be addressed through health policy or system interventions. For instance, electronic medical record reminder systems may be able to use recorded body mass index to remind providers to perform cancer screening for patients with obesity or to remind providers to offer indicated immunizations for patients that are only present to the healthcare system occasionally. Or health care payers could incentivize providing immunization by improving reimbursement for these services. Finally, continued research should examine how to increase uptake and efficiency of vaccination among all people, especially in groups prone to healthcare disparities, including patients with obesity.

While our search criteria also included immunization for human papilloma virus, there were no studies eligible by our criteria. While this immunization has only been available within the last 10 years and so has been studied less than influenza and pneumococcal vaccination, future studies should consider whether receipt of immunization is affected by patient obesity as has been shown in other immunization and cancer screening interventions.

Our findings should be interpreted within the limitations of our study design. The studies identified in our systematic review and meta-analysis demonstrates significant heterogeneity in the direction and effect size of the association between obesity and receipt of influenza and pneumococcal vaccination. The index of heterogeneity in this the meta-analysis ranges from 0 % (no heterogeneity between studies) to 96.4 % (high heterogeneity between studies) depending on the vaccination type and obesity comparison. The wide confidence intervals observed in our meta-analysis are attributable to effect size variations in the included studies. Varied study designs (high quality cross-sectional survey vs. administrative record studies), and differences in sample selection, measurement of vaccine receipt, and adjustment for possible confounding explains much of heterogeneity in effect direction as well as the broad confidence intervals noted in this meta-analysis.

In addition to heterogeneity in study populations, the included studies’ models vary significantly in the selection of covariates used to control for confounding. Beyond the importance of age, sex, and race for reasons noted above, controlling for health care utilization is important. Three of the nine studies controlled for medical care utilization (in either number of outpatient visits and/or hospitalizations in the past year). Increasing body mass index is associated with increased utilization of medical services [45, 46]. Increasing interactions with the healthcare system likely increase the chance of receiving preventive care services like vaccinations over time. By controlling for utilization, the results of the study better describe the odds of receipt of vaccination services during a single visit, rather than during a time interval such as the last 12 months, which is the outcome of interest in many of these studies. However, in the ideal situation where the patient receives the indicated vaccination at the first visit, controlling for all visits after this one dilutes the effect of the appropriate intervention because the outcome is controlled for total amount of utilization, not just utilization before vaccination.

## Conclusion

In summary, the receipt of vaccination for pneumococcus and influenza is an important health care quality indicator. While obese patients are associated with higher levels of vaccination, the overall levels of vaccination are low in all populations. Our findings highlight the importance of continued efforts to improve patient access to and utilization of recommended adult vaccinations. Persons with comorbidities like obesity are particularly vulnerable to morbidity from vaccine-preventable illnesses, and future research is needed to better identify patient-level and systems-level predictors of vaccine uptake in this group.

### Ethics approval and consent to participate

The study was deemed exempt from human subject research approval by The University of Michigan Medical Institutional Review Board (HUM00097519). The study did not involve human or animal tissue.

### Consent for publication

Not applicable

### Availability of data and materials

The data compiled and used for meta-analysis is available at https://zenodo.org/record/47856.

## Appendix 1

**Table 2:** Full search vocabulary and search results

Population PubMed MeSH (“anthropometry”[MeSH Terms] OR “body mass index”[MeSH Terms] OR “body weight”[MeSH Terms] OR “overweight”[MeSH Terms] OR “obesity”[MeSH Terms] OR “risk factors”[MeSH Major Topic] OR “life style”[MeSH Major Topic] OR “adiposity”[MeSH Terms]) PubMed Keywords (“body mass index” [tiab] OR “body weight” [tiab] OR “body mass” [tiab] OR “overweight” [tiab] OR “body fat” [tiab] OR “obesity” [tiab] OR “obese” [tiab] OR “heavy” [tiab] OR “risk factors” [tiab] OR “adiposity” [tiab] OR “body mass” [tiab]) EMBASE Emtree ‘anthropometric parameters’/exp OR ‘body mass’/exp OR ‘obesity’/exp OR ‘adiposity’/exp OR ‘overweight’/exp Keywords OR obesity OR body mass OR overweight OR obese OR risk factor OR adiposity Intervention PubMed MeSH “Immunization”[Mesh]OR “Vaccines”[Mesh] OR “Pneumococcal Infections”[Mesh] OR “Pneumococcal Vaccines”[Mesh] OR “Influenza Vaccines”[Mesh] OR “Influenza, Human/prevention and control”[Mesh] OR”Papillomavirus Vaccines”[Mesh] OR “Human papillomavirus 6”[Mesh] OR “Human papillomavirus 11”[Mesh] OR “Human papillomavirus 18”[Mesh] OR “Human papillomavirus 16”[Mesh] OR “Human papillomavirus 6”[Mesh] PubMed Keywords (“Immunization” [tiab] OR “Vaccination” [tiab] OR “Vaccine” [tiab] OR “pneumococcal vaccine” [tiab] OR “pneumonia vaccine” [tiab] OR “pneumococcal immunization” [tiab] OR “pneumonia immunization” [tiab]OR “influenza vaccine” [tiab] OR “influenza immunization” [tiab] OR “flu vaccine” [tiab] OR “HPV vaccine” [tiab] OR “human papillomavirus virus vaccine” [tiab] OR “HPV immunization” [tiab] OR “human papillomavirus virus immunization” [tiab] OR “Gardasil” [tiab] OR “Silgard” [tiab] OR “Cervarix” [tiab]) EMBASE Emtree ‘immunization’/exp OR ‘pneumococcal infection’/exp OR ‘influenza’/exp OR ‘papilloma virus’/exp Keywords (Immunization OR Vaccination OR Vaccine OR pneumococcal vaccine OR pneumonia vaccine OR pneumococcal immunization OR pneumonia immunization OR influenza vaccine OR influenza immunization OR flu vaccine OR HPV vaccine OR human papillomavirus virus vaccine OR HPV immunization OR human papillomavirus virus immunization OR Gardasil OR Silgard) Outcome PubMed MeSH (“Diagnostic Errors”[MeSH Terms]) OR PubMed Keywords “screening rate” [tiab] OR “immunization rate” [tiab] OR “vaccination rate” [tiab] OR “repeat screening” [tiab] OR “quality of care” [tiab] OR “preventive services” [tiab] OR “preventive care” [tiab] OR “medical utilization” [tiab] OR “receipt of screening” [tiab] EMBASE Emtree ‘diagnostic error’/exp OR ‘preventive health service’/exp OR ‘health care utilization’/exp Keywords ‘screening rate’ OR ‘immunization rate’ OR ‘vaccination rate’ OR ‘repeat screening’ OR ‘quality of care’ OR ‘preventive services’ OR ‘preventive care’ OR ‘medical utilization’ OR ‘receipt of screening’ Web of Science: Population, Intervention and Outcome TS = ((‘body mass’[TS] OR ‘obesity’ OR ‘overweight’ OR ‘obese’ OR ‘adiposity’) AND (Immunization OR Vaccination OR Vaccine OR pneumococcal vaccine OR pneumonia vaccine OR pneumococcal immunization OR pneumonia immunization OR influenza vaccine OR influenza immunization OR flu vaccine OR HPV vaccine OR human papillomavirus virus vaccine OR HPV immunization OR human papillomavirus virus immunization OR Gardasil OR Silgard) AND (screening rate OR immunization rate OR vaccination rate OR repeat screening OR quality of care OR preventive services OR preventive care OR medical utilization OR receipt of screening)) *Refined by: DOCUMENT TYPES: (PROCEEDINGS PAPER)* *Indexes = SCI-EXPANDED, CPCI-S Timespan = All years* Results of Search: PubMed Population = 1071513 10/28/2014 Intervention = 343246 10/28/2014 Outcome = 125015 10/28/2014 Population + Intervention + Outcome =134 10/28/2014 Population = 1115328 5/29/2015 Intervention = 352483 5/29/2015 Outcome = 128816 5/29/2015 Population + Intervention + Outcome =145 5/29/2015 EMBASE Population = 1535906 10/30/2014 Intervention = 339867 10/30/2014 Outcome = 162837 10/30/2014 Population + Intervention + Outcome = 642 10/30/2014 Population + Intervention + Outcome NOT [Medline]/lim = 161 10/30/2014 Population = 1638024 5/29/2015 Intervention = 353931 5/29/2015 Outcome = 172366 5/29/2015 Population + Intervention + Outcome = 773 5/29/2015 Population + Intervention + Outcome NOT [Medline]/lim = 187 5/29/2015 Web Of Science Population + Intervention + Outcome + only conference proceedings 8 10/31/2014 Population + Intervention + Outcome + only conference proceedings 8 5/29/2015	

## Appendix 2

**Table 3:** Summary of tests of heterogeneity and publication bias

Vaccination and obesity classification	I^2^ statistic	Begg’s test
Influenza vaccination, Obesity (>30 kg/m^2^)	70.3%	0.458
Influenza vaccination, Class I Obesity (30-34.9 kg/m^2^)	95.9%	0.327
Influenza vaccination, Class II Obesity (34-39.9 kg/m^2^)	94.9%	0.624
Influenza vaccination, Class III Obesity (>40 kg/m^2^)	96.4%	0.327
Pneumococcal vaccination, Obesity (>30 kg/m^2^)	0.0%	0.602
Pneumococcal vaccination, Class I Obesity (30-34.9 kg/m^2^)	75.2%	0.042
Pneumococcal vaccination, Class II Obesity (34-39.9 kg/m^2^)	15.1%	0.174
Pneumococcal vaccination, Class III Obesity (>40 kg/m^2^)	84.9%	0.042

## Additional file

Additional file 1: PRISMA 2009 Checklist. (DOCX 29 kb)

## Abbreviations

WHO: World Health Organization
SAGE: Strategic Advisory Group of Experts
US: United States of America
CDC: Centers for Disease and Control and Prevention
HIV: human immunodeficiency virus
PRISMA: Preferred Reporting Items for Systematic Reviews and Meta-Analyses
MOOSE: Guidelines for Meta-Analyses and Systematic Reviews of Observational Studies
BMI: body mass index
VA: Veteran’s Health Administration
NIH: National Institute of Health
NHLBI: National Heart, Lung and Blood Institute
